# CULTURAL DIMENSION OF THE DISCREPANCY OF PLANNED AND ACTUAL RETIREMENT AGE IN THE U.S.: HISPANICS VERSUS NON-HISPANICS

**DOI:** 10.1093/geroni/igz038.475

**Published:** 2019-11-08

**Authors:** Antonia E Diaz-Valdes Iriarte

**Affiliations:** Boston College School of Social Work, MA, United States

## Abstract

The sustainability problems to the SS has led to a glowing debate about what the full retirement age should be and if working longer is a plausible option for everyone or just for those who have some control over their retirement decisions (e.g., Munnell & Sass, 2008; McNamara & Williamson, 2013; Munnell et al, 2016). All ethno-racial groups have increased their average retirement age over the last years. However, Hispanics’ retirement age is still lower even if they stated they plan to continue to work at retirement (EBRI 2008; Diaz-Valdes, 2018). Most studies about retirement timing have focused on middle-class Whites, and the prediction of planned or actual retirement separately. One of the lesser studied complexities of the retirement conundrum concerns ethno-racial differences and cultural-related predictors of retirement timing (Lytle et al, 2015). This study seeks to extend the understanding of differences between Hispanics and non-Hispanics regarding the timing of retirement relative to when they thought they would retire by including a broad array of cultural and family related predictors. Multinomial regression models were used. The results indicate significant differences between Hispanics and non-Hispanic Whites. Taking care of grandchildren was a significant predictor among Hispanics but not among non-Hispanic Whites. For Hispanics taking care of grandchildren, for over 20 hrs., was associated with a decreased probability of stating they will never retire. The increase of one dependent was associated with an increased on the probability of retiring earlier than planned. The effect of one additional dependent was larger for non-Hispanics.

